# Fetal MRI deep learning segmentation of body and lung in congenital diaphragmatic hernia

**DOI:** 10.1093/radadv/umae034

**Published:** 2024-12-12

**Authors:** Leon M Bischoff, Sebastian Nowak, Maximilian Mader, Maike Theis, Thomas Vollbrecht, Alexander Isaak, Daniel Kuetting, Claus C Pieper, Annegret Geipel, Florian Kipfmueller, Brigitte Strizek, Alois M Sprinkart, Julian A Luetkens

**Affiliations:** Department of Diagnostic and Interventional Radiology, University Hospital Bonn, Bonn, NRW, 53127, Germany; Quantitative Imaging Lab Bonn (QILaB), University Hospital Bonn, Bonn, NRW, 53127, Germany; Department of Diagnostic and Interventional Radiology, University Hospital Bonn, Bonn, NRW, 53127, Germany; Quantitative Imaging Lab Bonn (QILaB), University Hospital Bonn, Bonn, NRW, 53127, Germany; Department of Diagnostic and Interventional Radiology, University Hospital Bonn, Bonn, NRW, 53127, Germany; Department of Diagnostic and Interventional Radiology, University Hospital Bonn, Bonn, NRW, 53127, Germany; Quantitative Imaging Lab Bonn (QILaB), University Hospital Bonn, Bonn, NRW, 53127, Germany; Department of Diagnostic and Interventional Radiology, University Hospital Bonn, Bonn, NRW, 53127, Germany; Quantitative Imaging Lab Bonn (QILaB), University Hospital Bonn, Bonn, NRW, 53127, Germany; Department of Diagnostic and Interventional Radiology, University Hospital Bonn, Bonn, NRW, 53127, Germany; Quantitative Imaging Lab Bonn (QILaB), University Hospital Bonn, Bonn, NRW, 53127, Germany; Department of Diagnostic and Interventional Radiology, University Hospital Bonn, Bonn, NRW, 53127, Germany; Quantitative Imaging Lab Bonn (QILaB), University Hospital Bonn, Bonn, NRW, 53127, Germany; Department of Diagnostic and Interventional Radiology, University Hospital Bonn, Bonn, NRW, 53127, Germany; Department of Obstetrics and Prenatal Medicine, University Hospital Bonn, Bonn, NRW, 53127, Germany; Department of Neonatology and Pediatric Critical Care Medicine, University Hospital Bonn, Bonn, NRW, 53127, Germany; Department of Obstetrics and Prenatal Medicine, University Hospital Bonn, Bonn, NRW, 53127, Germany; Department of Diagnostic and Interventional Radiology, University Hospital Bonn, Bonn, NRW, 53127, Germany; Quantitative Imaging Lab Bonn (QILaB), University Hospital Bonn, Bonn, NRW, 53127, Germany; Department of Diagnostic and Interventional Radiology, University Hospital Bonn, Bonn, NRW, 53127, Germany; Quantitative Imaging Lab Bonn (QILaB), University Hospital Bonn, Bonn, NRW, 53127, Germany

**Keywords:** fetal, magnetic resonance imaging (MRI), congenital diaphragmatic hernia (CDH), prognosis, nnU-Net

## Abstract

**Purpose:**

To determine if deep learning (DL) segmentation of total fetal body volume (TFBV) and total fetal lung volume (TFLV) in fetuses with congenital diaphragmatic hernia has comparable performance to manual segmentation.

**Materials and Methods:**

A total of 208 fetal MRI studies with congenital diaphragmatic hernia, acquired between August 2007 and September 2023, were retrospectively included. TFBV and TFLV were extracted from manual tissue segmentations in balanced gradient echo and single shot T2-weighted turbo spin echo sequences. MRI studies were split into training (n = 188) and hold-out test data (n = 20). Wilcoxon signed-rank test was used to compare manual and DL-based segmentations by 2 U-Nets. Manual and DL segmentation times were noted and compared using Student’s t-test. The observed/expected ratio of the total lung volume (O/E TLV) as a prognostic marker for postnatal survival was calculated. Outcome predictions of O/E TLV for postnatal death were assessed with univariate regression analysis.

**Results:**

Manual segmentation times were higher compared to DL segmentations (30 ± 7 minutes versus 0.25 ± 0.05 minutes, *P *<* *.001). Manual and DL-based TFBV were similar (1317 ± 498 mL versus 1306 ± 491 mL; *P* = .04; Dice score: 0.98 ± 0.01). TFLV (19.4 ± 11.5 mL versus 18.7 ± 12.4 mL; *P = *.11; Dice score: 0.84 ± 0.09) and O/E TLV (39.3 ± 18.1 mL versus 37.7 ± 19.1 mL, *P = *.13) were not significantly different. Postnatal mortality was negatively associated with higher manual O/E TLV (odds ratio: 0.97; 95% confidence interval [CI], 0.96–0.98; *P *<* *.001) and DL O/E TLV (odds ratio: 0.97; 95% CI, 0.96–0.98; *P *<* *.001).

**Conclusion:**

DL for body and lung segmentation in fetuses with congenital diaphragmatic hernia allows reliable and rapid calculations of the observed/expected ratio and equally predicts prognostic outcome.

**Abbreviations**

CDH = congenital diaphragmatic hernia; CI = confidence interval; DL = deep learning; ECMO = extracorporeal membrane oxygenation; GRE = gradient echo; O/E TLV = observed/expected ratio of total lung volume; T2w = T2-weighted; TFBV = total fetal body volume; TFLV = total fetal lung volume; TSE = turbo spin echo sequence.

**Summary**

In fetal MRIs with congenital diaphragmatic hernias, deep learning segmentation calculated lung volumes similar to manual segmentation, but in a much shorter time (15 seconds versus 30 minutes, respectively).

**Key Results**

- Deep learning (DL) segmentation of body and lung volumes on fetal MRI with congenital diaphragmatic hernias had Dice scores of 0.98 ± 0.01 and 0.84 ± 0.09 in the training and validation cohorts, respectively.
- As a prognostic marker for postnatal survival, manual and DL observed/expected of the total lung volume ratios (39.3 ± 18.1% versus 37.7 ± 19.1%, *P = *.13) were comparable.
- Time required for DL segmentation was less than a minute.

## Introduction

Congenital diaphragmatic hernia (CDH) is a rare disease characterized by pulmonary hypoplasia following a defect of the diaphragm and herniation of abdominal organs into the thorax. This often results in postnatal pulmonary hypertension and hypoxemia after birth.^1^ Approximately 2–3 in 10 000 births are affected with an overall mortality of 38%.^2–4^ Although establishing the diagnosis is usually done by prenatal ultrasound, fetal MRI is employed in addition to ultrasound for estimation of total fetal body volumes (TFBV) and lung volumes (TFLV) to make prognostic statements about fetal viability.^5–7^ Dependent on gestational age, TFBV, and TFLV, a variety of reference values for the expected fetal lung volume and the observed/expected ratio of total lung volume (O/E TLV) were established.^8^^,^^9^ Accurate estimates of TFBV and TFLV are vital for reliable calculation of O/E TLV, as a low O/E TLV is a measure of severe pulmonary hypoplasia.^6^ This may be a direct indication for fetal endoluminal tracheal occlusion and can reduce the risk of postnatal death and the necessity of extracorporeal membrane oxygenation (ECMO).^10^ In particular, an O/E TLV of <25% is considered as indicative of a poor prognosis with survival rates of 0%–25%.^11^

However, manual segmentation of the fetal body and lung is time consuming, even for specialized radiologists, for 2 main reasons. First, reliable segmentation results require thin slice MRI acquisition, thus often more than 40 slices must be acquired for whole depiction of the fetus. Second, the anatomy is highly variable due to different degrees of viscus herniation or additional congenital anomalies.

Different deep learning (DL) algorithms using convolutional neural networks, most with the prominent U-Net architecture, have been developed and refined to efficiently segment the region of interest in medical images (eg, cardiac chambers or brain tumors).^12–15^ Although these algorithms have been applied to segmentation of fetal brain MRI, their application in other fetal organs is sparse due to their comparably smaller size and high anatomical variability in different developmental stages.^16^^,^^17^ Initial studies focusing on whole body and lung segmentation have shown good results, but only included images from healthy fetuses.^18^^,^^19^ Based on this, specific protocols for data annotation and neural network training for segmentation of fetuses with CDH have been proposed.^20^ However, advanced DL techniques for both whole body and lung segmentation in fetuses with CDH have not been thoroughly evaluated yet.

The aim of our study was to assemble a comprehensive dataset, annotated by experts, comprising MRI scans from fetuses with CDH and to develop and evaluate DL algorithms for automatic fetal body and lung segmentation. The primary aim of this study was to evaluate DL segmentation of TFBV and TFLV for the calculation of O/E TLV. The secondary aim was to assess the prognostic value of manual and DL O/E TLV for the prediction of ECMO and death.

## Materials and methods

### Study population

**Figure 1.**
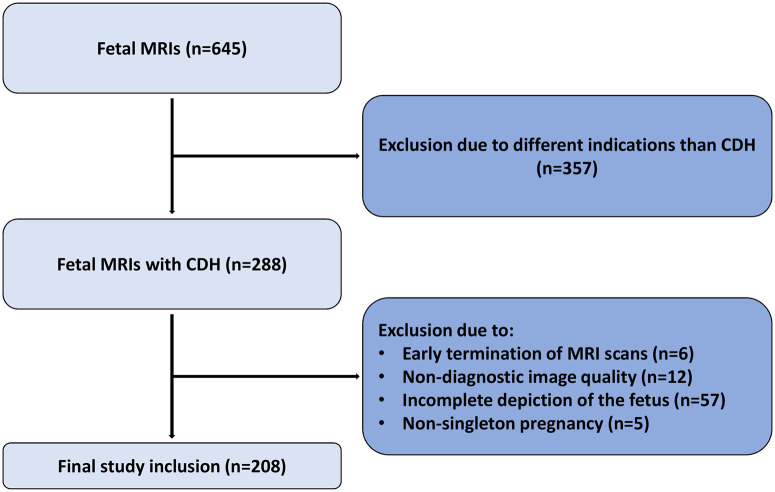
Inclusion and exclusion criteria of fetal MRIs with congenital diaphragmatic hernia (CDH).

The institutional review committee approved this retrospective single-center study. Because of the retrospective nature of the study, the requirement for written informed consent was waived. All patients who underwent fetal MRI between August 2007 and September 2023 in routine clinical workup were identified in the clinical data systems of the University Hospital Bonn. All fetuses with CDH, defined as hypoplasia of the lung from herniation of abdominal organs through a diaphragmatic defect, were included. Patients with pathologies that were not CDH, early termination of a fetal MRI, nonsingleton pregnancy, or suboptimal image quality (eg, fetal motion) were excluded from the study (Figure 1). Clinical parameters, including subsequent necessity of ECMO and death were assessed.

### Fetal MRI protocol

All images were acquired at 1.5 Tesla MRI systems (Ingenia 1.5 T or Intera 1.5 T, Philips Healthcare). The imaging protocol consisted of a balanced gradient echo (GRE) sequence for assessment of the fetal body and a T2-weighted (T2w) turbo spin echo sequence (TSE) for evaluation of the fetal lung. An additional T1-weighted balanced GRE sequence was acquired for assessment of meconium but was not used for the subsequent segmentation process. Detailed acquisition parameters are found in Table S1.

### Manual segmentation

**Figure 2.**
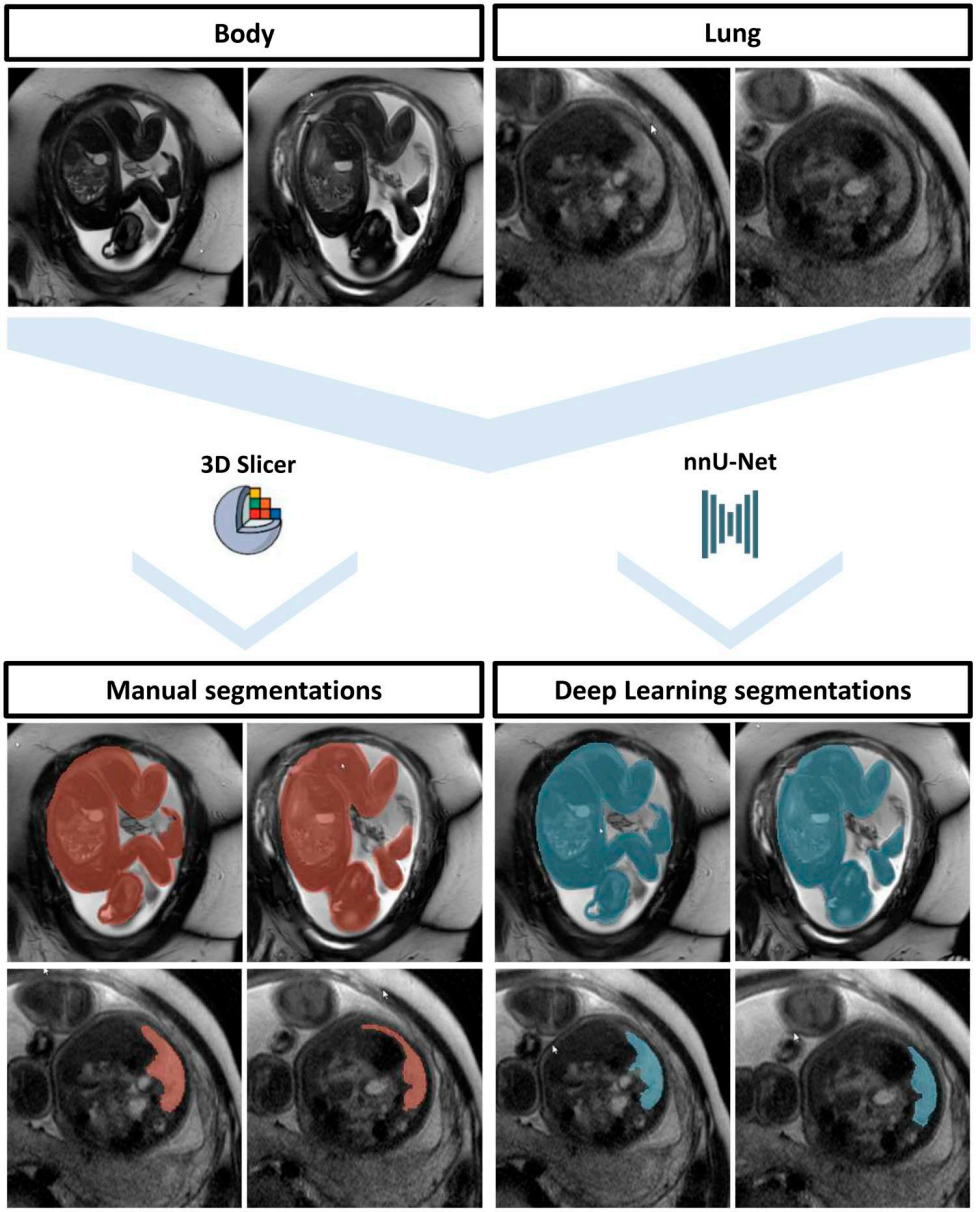
Illustration of the segmentation workflow for both manual and deep learning segmentations. Although the fetal body can be relatively easily delineated from the surrounding placental tissue, the differentiation of the T2-hyperintense lung tissue (arrows) from especially mediastinal structures is more complex. 3D Slicer was used for manual segmentations, whereas 2 separate 3D nnU-Net was trained for deep learning segmentations of fetal bodies and lungs.

Preferably, the sagittal balanced GRE sequence (body) and the axial T2w TSE sequence (lung) were used for segmentations of TFBV and TFLV, respectively, as these depicted the whole fetal body and lung in most patients. In some cases, different planes had to be used due to severe movement artifacts or incomplete depiction of the body/lung in the sagittal balanced GRE or axial T2w TSE sequence. All sequences were segmented with the open-source software 3D Slicer by either M.M. (1 year of experience in fetal MRI) or L.M.B. (3 years of experience in fetal MRI). To reduce potential inter-rater variability, all segmentations were double checked and, if necessary, adjusted by a board-certified radiologist with 11 years of experience in fetal MRI (J.A.L.).^21^ In cases of unclear tissue borders, additional sequences (balanced GRE and T2w TSE) in different planes were examined to clarify the fetal anatomy. However, only 1 balanced GRE and 1 T2w TSE sequence per fetus was ultimately annotated and used for training the DL algorithms (Figure 2).

### Deep learning algorithms

**Figure 3.**
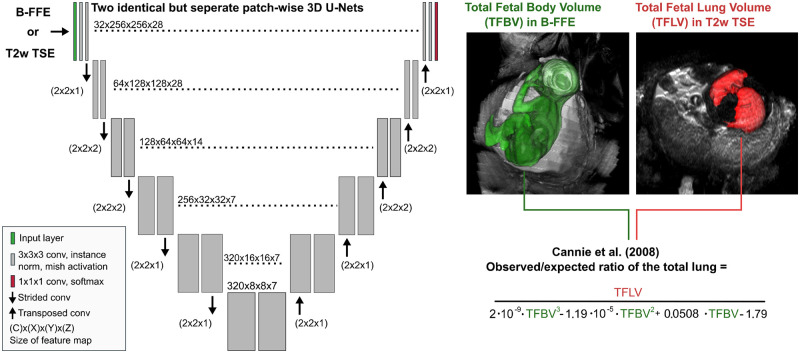
Illustration of the patch-wise 3D U-Nets trained for the total fetal body volume (TFBV) segmentation based on the balanced gradient echo (GRE) sequence and the total fetal lung volume (TFLV) segmentation based on T2-weighted turbo spin echo (T2w TSE) sequence. The TFBV and TFLV segmentations of the U-Nets are evaluated for determining the observed/expected ratio of the total lung volume according to Cannie et al.^6^.

To develop and evaluate DL segmentation of TFBV and TFLV, the data were randomly and without prior stratification for specific disease factors split into a hold-out test set and a training set with 5-fold cross-validation. Two patch-wise 3D U-Nets with a patch size of 256 × 256 × 28 were trained with 5-fold cross-validation for TFBV segmentation in sagittal balanced GRE and for TFLV segmentation in the axial T2w TSE using the nnU-Net framework.^22^ The patches for body segmentation had a resolution of 1.4 × 1.4 × 4.4 mm^3^ and for lung segmentation 1.2 × 1.2 × 3.3 mm^3^. The U-Net was extended to include Mish activation, was optimized by stochastic gradient descent with a Nesterov momentum of 0.99, poly learning rate scheme decreasing from 0.01, batch size of 2 patches, and oversampling of the foreground voxels.^23^ During training, images were randomly augmented by rotation, scaling, mirroring, brightness/contrast/gamma modification, Gaussian noise, blurring, and low-resolution simulation. Models were trained on a NVIDIA RTX 3090 GPU. To ensure convergence the TFBV segmentation was trained for 1000 epochs and the TFLV segmentation for 3000 epochs. Figure 3 illustrates models in detail.

### Statistical analysis

SPSS (IBM, version 27) was used for statistical analysis. Continuous variables are given as mean ± standard deviation and categorical variables as absolute numbers and percentages as appropriate. Dice scores for both TFBV and TFLV were calculated to evaluate the DL segmentations. Manual and DL segmentation times of the hold-out test dataset were noted and compared using Student’s t-test. The O/E TLV was calculated according to Cannie et al.^8^ Paired Student’s t-test was used for group comparisons of manual and DL segmentations for TFBV and TFLV, and for the comparison of O/E TLV. The means of their absolute and relative differences were calculated (ΔTFBV, ΔTFLV, and ΔO/E TLV). Linear regression analysis with Pearson correlation coefficient (r) was used for assessing the correlation between manual and DL segmentations of TFBV, TFLV, and O/E TLV, whereas Bland-Altman analysis was used for analysis of systematic differences. Odds ratios with 95% confidence intervals (CI) of both ECMO and death for both manual and DL segmentations were calculated for the TFBV, TFLV, O/E TLV, and birth weight by univariable binary logistic regression analysis. *P* values <.05 were considered statistically significant.

### Code availability

The codes for training and testing of the nnU-Net algorithm are found under: https://github.com/MIC-DKFZ/nnUNet/tree/nnunetv1. The specific training parameters are found under the deep learning algorithms section.

## Results

### Clinical characteristics

A total of 645 fetal MRI studies were identified. Of those, 437 met exclusion criteria, resulting in inclusion of 208 fetal MRIs from 204 distinct fetuses (mean age of mother, 30 ± 6 years; mean gestational age, 28.9 ± 2.4 weeks). In 85% (177/208) of fetuses, CDH was present solely on the left side, in 91% (190/208) fetuses had remaining lung tissue on both sides, and in 60% (125/208) a liver-up constellation was present. A total of 22% (46/208) of fetuses died during pregnancy or postnatally on the intensive care unit. A detailed description of clinical parameters and outcome is given in Table 1.

**Table 1.** Clinical patient characteristics and outcome of 208 fetal MRIs from 204 distinct fetuses.

Variable	Value
Age mother (years)	30 ± 6
Gestational age (weeks)	28.9 ± 2.4
Biparietal diameter (mm)	77 ± 7
Head circumference (mm)	273 ± 23
Abdominal circumference (mm)	236 ± 26
Femur length (mm)	54 ± 6
Anhydramnios/oligohydramnios	4 (2)
Polyhydramnios	81 (39)
Liver-up	125 (60)
*CDH position*	
Only left	177 (85)
Only right	28 (13)
Both sides	3 (1)
*Remaining lung*	
Only left	5 (2)
Only right	11 (5)
Bilateral	192 (92)
Abortion	2 (1)
Cesarean section	119 (57)
Stillbirth	5 (2)
Postnatal death	41 (20)
Birth weight (g)	2840 ± 635
Apgar score at 1 minute	6 [IQR: 5–7]
Apgar score at 5 minutes	8 [IQR: 6.5–9]
Apgar score at 10 minutes	8 [IQR: 7–9]
ECMO	58 (28)
FETO	46 (22)

Continuous data are presented as mean ± standard deviation, ordinal data as median with interquartile range in square brackets, and categorical data as number of patients with percentages in parentheses.

Abbreviations: CDH = congenital diaphragmatic hernia, ECMO = extracorporeal membrane oxygenation, FETO = fetoscopic endoluminal tracheal occlusion, IQR = interquartile range.

### Training deep learning-based segmentation of fetal body and lung volumes

**Figure 4.**
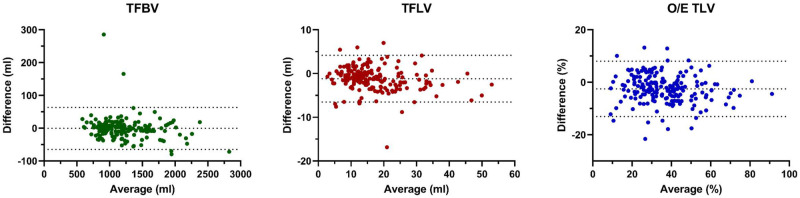
Comparison of manual and deep learning (DL) segmentations for the cross-validation dataset (n = 188). Bland-Altman plots show differences between manual and DL segmentations for the total fetal body volume (TFBV), total fetal lung volume (TFLV), and the observed/expected ratio of the total lung volume (O/E TLV) with mean relative differences of 1.5 ± 2.2 mL, 12.3 ± 11.6 mL, and 12.0 ± 9.8%, respectively. These differences are clearly reflected by fewer outliers in the DL TFBV segmentations, indicating excellent segmentation results for the latter. Overall, the calculated DL O/E TLV values resemble closely the DL TFLV values, thus the error in O/E TLV can be mostly attributed to the TFLV errors.

In the 5-fold cross-validation dataset (n = 188), mean TFBV showed no differences for the manual and DL segmentations (manual: 1213 ± 378 mL vs. DL: 1212 ± 372 mL; *P = *.82; Dice score: 0.98 ± 0.02). The mean TFLV differed between the manual and the DL segmentations (manual: 17.5 ± 9.4 mL vs. DL: 16.3 ± 8.8 mL; *P *<* *.001; Dice score: 0.82 ± 0.11). Mean ΔTFBV and ΔTFLV were 18.3 ± 27.1 mL and 2.2 ± 2.0 mL, respectively. The calculated O/E TLV for manual segmentations was slightly higher than for DL segmentations (manual: 38.3 ± 17.3% vs. DL: 35.7 ± 16.5%, *P *<* *.001) (Figure 4). Mean ΔO/E TLV was 4.6 ± 3.7%. See Table S2 for specific results.

### Hold-out test evaluation

**Figure 5.**
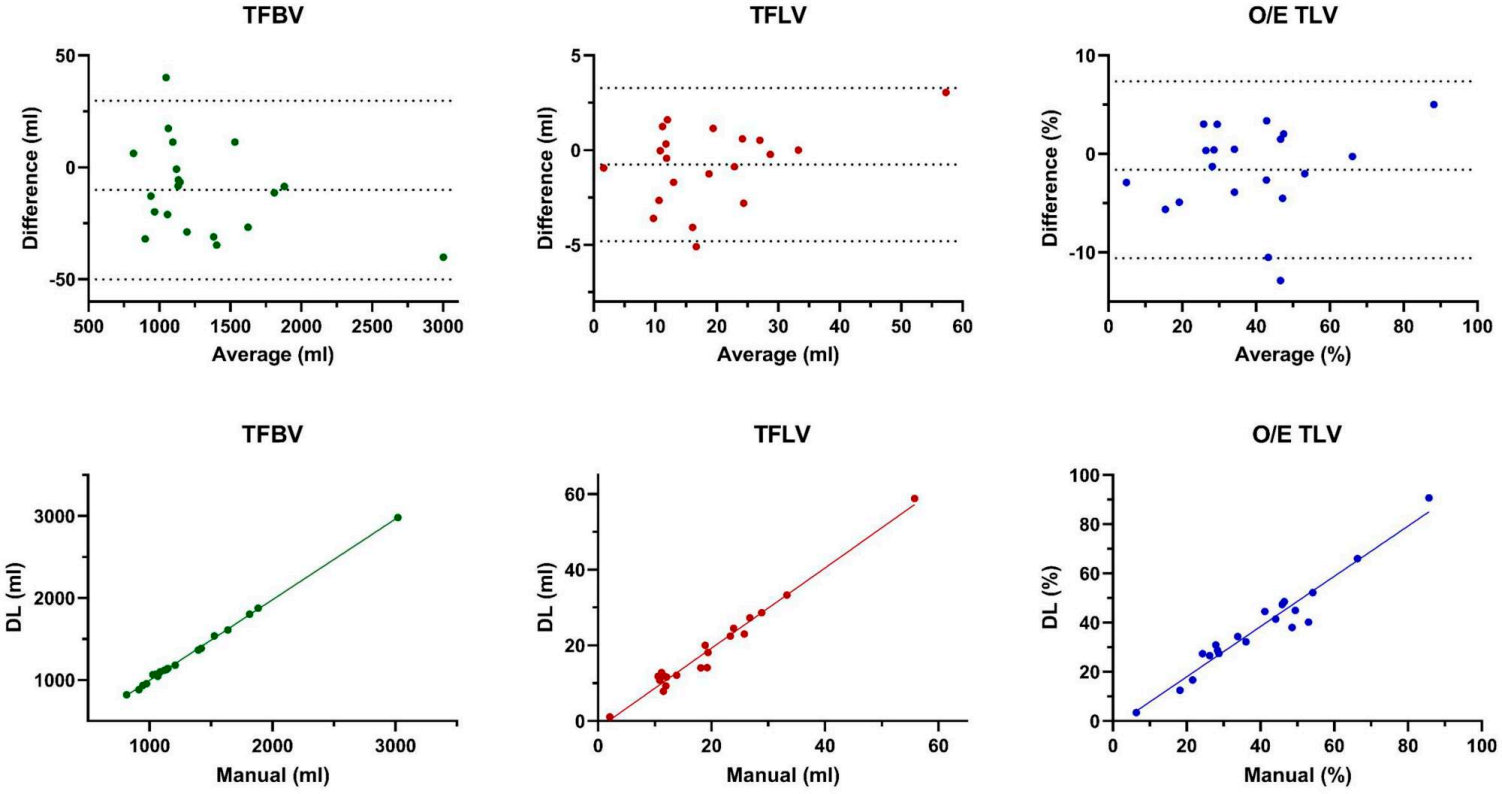
Comparison of manual and deep learning (DL) segmentations for the hold-out dataset (n = 20). Bland-Altman plots (top row) show differences between manual and DL segmentations for the total fetal body volume (TFBV), total fetal lung volume (TFLV), and the observed/expected ratio of the total lung volume (O/E TLV) with mean relative differences of 1.4 ± 0.9 mL, 8.3 ± 7.5 mL, and 9.0 ± 8.3%, respectively. The distribution is similar to the cross-validation dataset (Figure 4). However, the relative differences in the hold-out dataset are slightly smaller than in the latter, indicating good generalizability of the algorithms. Although the best correlation of manual and DL segmentations (bottom row) was found in the TFBV segmentations (Pearson coefficient, r = 0.999), the correlation for the TFLV segmentation (r = 0.988) and O/E TLV values (r = 0.971) were still excellent.

In the hold-out test dataset (n = 20), mean manual TFBV was slightly higher than mean DL TFBV (manual: 1317 ± 498 mL vs. DL: 1306 ± 491 mL; *P = *.04, Dice score: 0.98 ± 0.01). Manual and DL TFBV values were strongly correlated (r = 0.999; 95% CI, 0.996–1.0; *P *<* *.001). The mean TFLV of the manual and the DL segmentation was equivalent (manual: 19.4 ± 11.5 ml vs. DL: 18.7 ± 12.4 mL; *P *<* *.11; Dice score: 0.84 ± 0.09) and correlation of manual and DL TFLV values was high (r = 0.988; 95% CI, 0.941–0.996; *P *<* *.001). Mean ΔTFBV and ΔTFLV were 18.8 ± 12.3 mL and 1.6 ± 1.5 mL, respectively. The calculated O/E TLV for manual segmentations was equivalent to the DL segmentations (manual: 39.3 ± 18.1% vs. DL: 37.7 ± 19.1%, *P = *.13) with a mean ΔO/E TLV of 3.5 ± 3.3% (Figure 5). Correlation between manual and DL O/E TLV was high (r = 0.971; 95% CI, 0.910–0.992; *P *<* *.001). Manual segmentation times were higher compared to DL segmentations (30 ± 7 minutes versus 0.25 ± 0.05 minutes, *P *<* *.001). Specific results for type and location of hernias are listed in Table 2; segmentation examples in Figure S1.

**Table 2.** Results of the hold-out test dataset.

Parameter		Number	Manual	DL	Absolute Difference	Relative Difference	*P*-value
TFBV (mL)	All	20 (100)	1317 ± 498	1306 ± 491	18.8 ± 12.3	1.4 ± 0.9	.04
	Polyhydramnios	11 (55)	1442 ± 627	1431 ± 618	15.6 ± 11.6	1.1 ± 0.8	.07
	Anhydramnios/oligohydramnios	0 (0)	–	–	–	–	–
TFLV (mL)	All	20 (100)	19.4 ± 11.5	18.7 ± 12.4	1.6 ± 1.5	8.3 ± 7.5	.11
	Liver-down	9 (45)	24.4 ± 13.4	24.8 ± 14.1	1.5 ± 1.1	6.3 ± 4.5	.64
	Liver-up	11 (55)	15.2 ± 8.2	13.7 ± 8.5	1.8 ± 1.8	12.2 ± 11.6	.048
	CDH right side	1 (5)	11.5	7.9	3.6	31.3	–
	CDH left side	19 (95)	19.9 ± 11.7	19.2 ± 12.4	1.5 ± 1.4	7.6 ± 7.2	.20
	CDH both sides	0 (0)	–	–	–	–	–
	Lung parenchyma only right	0 (0)	–	–	–	–	–
	Lung parenchyma only left	0 (0)	–	–	–	–	–
	Lung parenchyma on both sides	20 (100)	19.4 ± 11.5	18.7 ± 12.4	1.6 ± 1.5	9.5 ± 9.1	.11
O/E TLV (%)	All	20 (100)	39.3 ± 18.1	37.7 ± 19.1	3.5 ± 3.3	9.0 ± 8.3	.13
	Polyhydramnios	11 (55)	37.2 ± 23.2	35.3 ± 24.9	3.5 ± 3.1	9.5 ± 8.2	.18
	Anhydramnios/oligohydramnios	0 (0)	–	–	–	–	–
	Liver-down	9 (45)	48.8 ± 18.5	49.6 ± 19.4	3.2 ± 2.1	6.6 ± 4.2	.57
	Liver-up	11 (55)	31.2 ± 14.1	28.1 ± 13.0	4.3 ± 4.1	13.7 ± 13.1	.07
	CDH right side	1 (5)	18.2	12.6	5.6	30.8	–
	CDH left side	19 (95)	40.4 ± 17.9	39.1 ± 18.6	3.4 ± 3.3	8.5 ± 8.2	.20
	CDH both sides	0 (0)	–	–	–	–	–
	Lung parenchyma only right	0 (0)	–	–	–	–	–
	Lung parenchyma only left	0 (0)	–	–	–	–	–
	Lung parenchyma on both sides	20 (100)	39.3 ± 18.1	37.7 ± 19.1	3.5 ± 3.3	9.0 ± 8.3	.13

The O/E TLV was calculated after Cannie et al.^6^ Continuous data are presented as mean ± standard deviation and categorical data as number of patients with percentages in parentheses. The absolute difference was defined as the direct difference of manual and DL values. The relative difference was defined as the percental differences of manual and DL values.

Abbreviations: DL = deep learning, O/E TLV = observed/expected ratio of the total lung volume, TFBV = total fetal body volume, TFLV = total fetal lung volume.

### Outcome predictors

**Figure 6.**
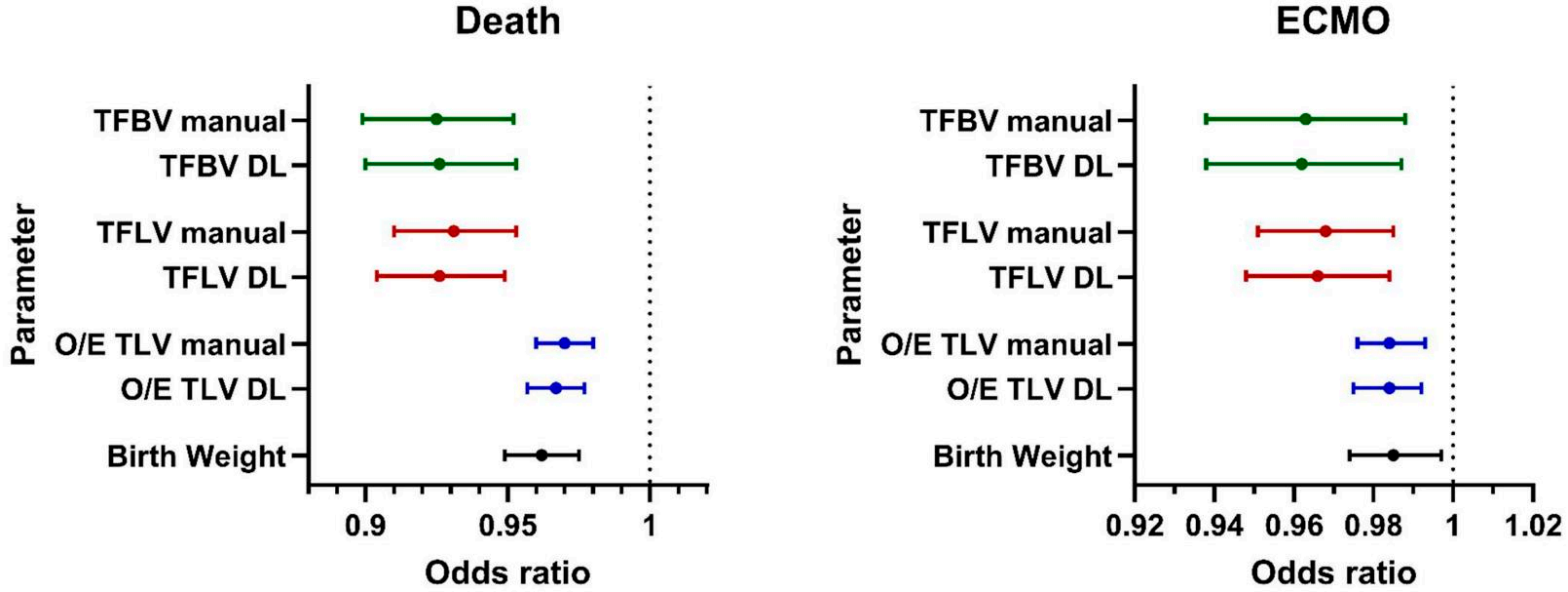
Forest plots for odds ratios of death and extracorporeal membrane oxygenation (ECMO). All segmentation parameters, including the total fetal body volume (TFBV), total fetal lung volume (TFLV), and the calculated observed/expected ratio of the total lung volume (O/E TLV) for both manual and deep learning (DL) segmentations reduced the risk of death and ECMO. Similar results were found for the birth weight. 95% confidence intervals of each odds ratio are shown.

Considering the whole dataset, an O/E TLV of <25% for manual and DL segmentations was found in 21% (43/208) and 23% (47/208) of fetuses, respectively. Higher values of manual measured TFBV, TFLV, and the calculated O/E TLV were associated with a reduced risk of both ECMO (range odds ratio: 0.96–0.98; 95% CI, 0.94–0.99; *P = *<.001–.004) and death (range odds ratio: 0.93–0.97; 95% CI, 0.90–0.98; *P *<* *.001). DL extracted values for TFBV, TFLV, and the calculated DL O/E TLV were similarly associated with a lower risk of ECMO (range odds ratio: 0.96–0.98; 95% CI, 0.94–0.99; *P* = <.001–.01) and death (range odds ratio: 0.93–0.97; 95% CI, 0.90–0.98; *P *<* *.001). Additionally, a higher birth weight was associated with a reduced risk of ECMO (odds ratio: 0.985; 95% CI, 0.974–0.997; *P *≤* *.001) and death (odds ratio: 0.962; 95% CI, 0.949–0.975; *P *≤* *.001) (Figure 6 and Figure S2).

## Discussion

The aim of our study was to assemble an extensive dataset of fetal MRI with confirmed CDH and use it to train DL algorithms based on nnU-Net for automatic segmentation of fetal body and lung volumes. We found our algorithm produced accurate segmentations of the TFBV in our hold-out dataset, only differing by about 1.4 ± 0.9% from manual segmentations. Although TFLV (8.3 ± 7.5%) and ultimately the O/E TLV (9.0 ± 8.3%) had higher relative differences, DL-calculated TFBV, TFLV, and O/E TLV demonstrated equal odds ratios for death and for the necessity of ECMO, while not requiring human annotation and being 120 times faster.

In contrast to previous studies investigating fetal body segmentation, we included a large cohort of fetuses with CDH.^24^^,^^25^ However, segmentation of the TFBV was challenging because of several factors. Not only did the fetus size range from 571 to 3022 mL, but also the clear differentiation of fetal from placental tissue was complicated by different amounts of amniotic fluid with a poly- or anhydramnios being present in 41% of cases. Interestingly, after stratification for either an- and oligohydramnios or polyhydramnios, the agreement between segmentations was still excellent in the cross-validation dataset. In the hold-out dataset, only fetuses with normal amounts of amniotic fluid or polyhydramnios, but not an-/oligohyramnios were found due to random distribution of cases. However, the DL algorithm showed an equally excellent performance in this subgroup, indicating high adaptability of the DL algorithm to different anatomies. Overall, despite manual and DL TFBV segmentations were statistically different (*P *= .04), mean relative difference was small with 1.4 ± 0.9% and thus this difference is negligible in clinical practice.

Because of the high heterogeneity of CDH, affected fetuses have highly variable anatomy of both lungs. For instance, although only 16% of hernias occur on the right side, it is associated with a worse prognosis.^26–28^ Our DL algorithm trained on 208 fetal MRIs achieved a Dice score of 0.84 ± 0.09 in the hold-out dataset for segmentation of the TFLV (Dice score of cross-validation dataset: 0.82 ± 0.11). Although this is lower compared to the results for the TFBV segmentation, this can be attributed to the more difficult differentiation of lung tissue from surrounding fetal tissue and the comparably lower absolute number of voxels. Therefore, the inclusion of a single additional voxel has a much greater impact on the segmentation results. Because of its overall rarer occurrence, the lung segmentation of fetuses with right-sided CDH was more difficult for the DL algorithm, resulting in higher mean differences in the cross-validation dataset (this subgroup was underrepresented in the hold-out dataset because of random case allocation at the study outset). Additional inclusion of data with right-sided CDH could further strengthen the algorithm, whereas separate algorithms would be desirable if datasets are balanced. The overall more variable segmentation results of TFLV would necessitate a careful visual inspection and possibly an adjustment of DL segmentation results. This, however, would still be significantly faster than traditional manual segmentation.

The calculation of the O/E TLV is influenced by both the TFBV and TFLV.^8^ However, the relative mean differences of the manual and DL O/E TLV closely resembled the mean differences of the TFLV, reflecting the high accuracy of the TFBV segmentations. Although a relative difference of 9.0 ± 8.3% between manual and DL O/E TLV was observed, the prognostic value was equal. Furthermore, postnatal outcome analysis of DL segmentations with death and ECMO were exceedingly similar not only to the reference standard of manual segmentations, but also in accordance with prior studies.^29^^,^^30^

In clinical practice, DL segmentations could be used for an initial estimate of postnatal prognosis, simultaneously saving clinicians valuable time. However, because the potential outcome parameters, including death and ECMO, have high impact on both the fetus and any other involved individual like parents and health care professionals, definite statements about TFBV, TFLV, and O/E TLV in the final report must be made by the reporting radiologist. Overall, a clinical adaption of this algorithm could not only reduce segmentation time, but more importantly could compensate for reader inexperience, as the algorithm can reliably segment most types and extents of CDH. However, before this algorithm can be used in real-world scenarios, additional training and validation on data from different sites needs to be included to tackle complex anatomical variations.

Our study has limitations. First, we included only MRIs from 2 scanner types from a single manufacturer. To both increase the reliability of the algorithm and to further validate the results, annotated multicenter MRI data with a variety of scanners should be included in further studies. Transfer learning could help to accelerate the training process. An implementation of the DL algorithm across multiple centers with varying MRI protocols might be challenging. However, differences in scanner resolution or patient positioning would strengthen the algorithm, as it exposes it to greater variability. Second, although our method had an overall good performance for segmentation of the TFBV, the high anatomical variance of CDH necessitates even larger datasets for reliable segmentations in more complex cases. Because of the low incidence of this disease, multicenter studies are required. Third, not all subgroups of our patient collective were present in our hold-out dataset because of the initial random allocation of cases among the test and hold-out datasets. Although this was done to handle selection bias, it also restricts interpretation of rare anatomical variants. This aspect would also be addressed by larger datasets across multiple sites. Fourth, as this study explicitly excluded nonsingleton pregnancies, this population is not represented in the dataset. However, as in our clinic only 5 cases occurred in 16 years, the benefit of DL automation in nonsingleton pregnancies is limited. Fifth, all prognostic studies in fetal CDH face inherent limitations due to the complex, multifactorial nature of the condition and its outcomes. Thus, while relative lung volumes provide important information about the degree of pulmonary hypoplasia, they are not the sole predictors of postnatal outcomes that is also influenced by eg, cardiac dysfunction and pulmonary hypertension.

In conclusion, in MRI of fetuses with CDH, we achieved excellent Dice scores for the automatic body segmentation, whereas Dice scores for the fetal lung segmentation were slightly worse because of high anatomical variations of the lung tissue. Time required for DL segmentation was less than 1 minute. However, the O/E TLV as a prognostic measure was equal between manual and DL segmentations and had an equal prognostic value, including prediction of death and extracorporeal membrane oxygenation.

## Supplementary Material

umae034_Supplementary_Data

## Data Availability

The artificial intelligence algorithms and training parameters underlying this article are available in the article and at https://github.com/MIC-DKFZ/nnUNet/tree/nnunetv1. Further data underlying this article will be shared on reasonable request to the corresponding author.
