# Supplementary material for: Fetal MRI deep learning segmentation of body and lung in congenital diaphragmatic hernia
Source: Radiol Adv. 2024 Dec 12;2(1):umae034. doi: 10.1093/radadv/umae034 (PMC12429181; doi:10.1093/radadv/umae034)
Supplement: umae034_Supplementary_Data [file umae034_Supplementary_Data.zip › Supplement.pdf]

Supplemental

Fetal MRI deep learning segmentation of body and lung in congenital diaphragmatic hernia

*Leon M. Bischoff, MD[1,2], *Sebastian Nowak, PhD[1,2], Maximilian Mader[1], Maike Theis, M.Sc.[1,2], Thomas Vollbrecht, MD[1,2], Alexander Isaak, MD[1,2], Daniel Kuetting, MD[1,2], Claus C. Pieper, MD[1], Annegret Geipel, MD[3], Florian Kipfmueller, MD[4], Brigitte Strizek, MD[3], *Alois M. Sprinkart, PhD[1,2], *Julian A. Luetkens, MD[1,2]

*contributed equally

[1]Department of Diagnostic and Interventional Radiology, University Hospital Bonn, Bonn, NRW, Germany
[2]Quantitative Imaging Lab Bonn (QILaB), University Hospital Bonn, Bonn, NRW, Germany
[3]Department of Obstetrics and Prenatal Medicine, University Hospital Bonn, Bonn, NRW, Germany
[4]Department of Neonatology and Pediatric Critical Care Medicine, University Hospital Bonn, Bonn, NRW, Germany

Corresponding author:
Julian A. Luetkens, MD
Department of Diagnostic and Interventional Radiology
University Hospital Bonn
Venusberg-Campus 1
53127 Bonn
Germany
Phone: +49 (0)228-287-11831
Fax: +49 (0)228-287-15598
Email: julian.luetkens@ukbonn.de

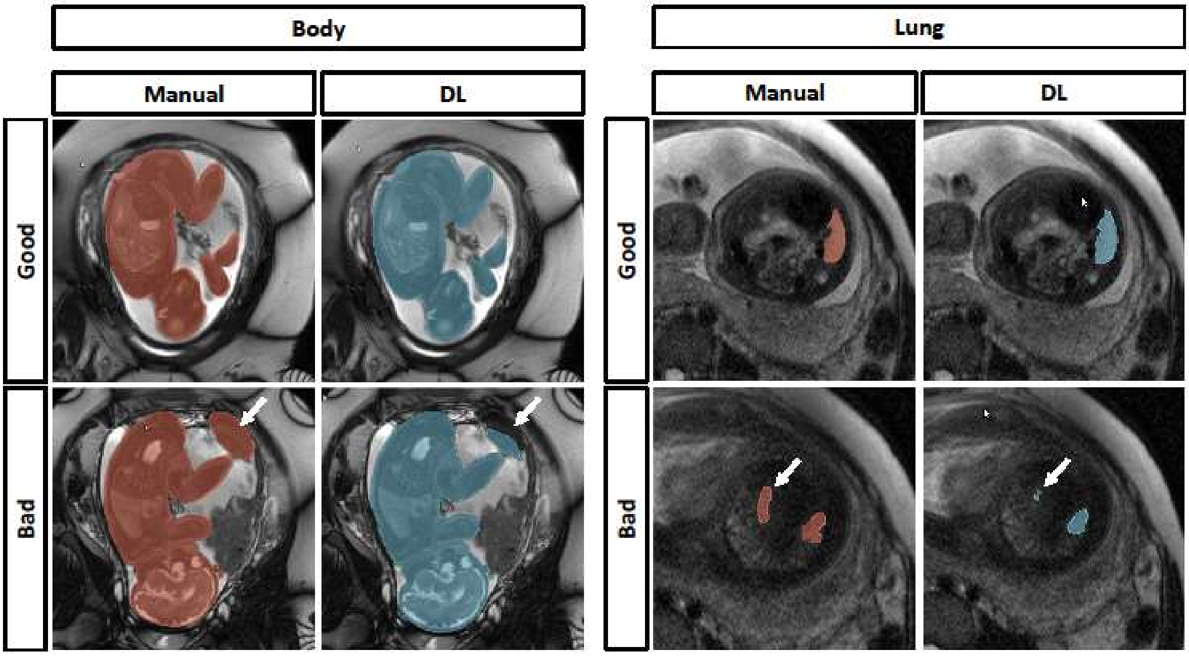

Figure S1: Examples of good and bad deep learning (DL) segmentations compared to manual segmentations. White arrows mark insufficient DL segmentation results. While the example for the bad body segmentation shows an incomplete segmentation of one fetal foot, the example for the bad lung segmentation shows incomplete segmentation of the left fetal lung. In contrast, good segmentation examples show matching results with only minimal differences for both body and lung segmentations.

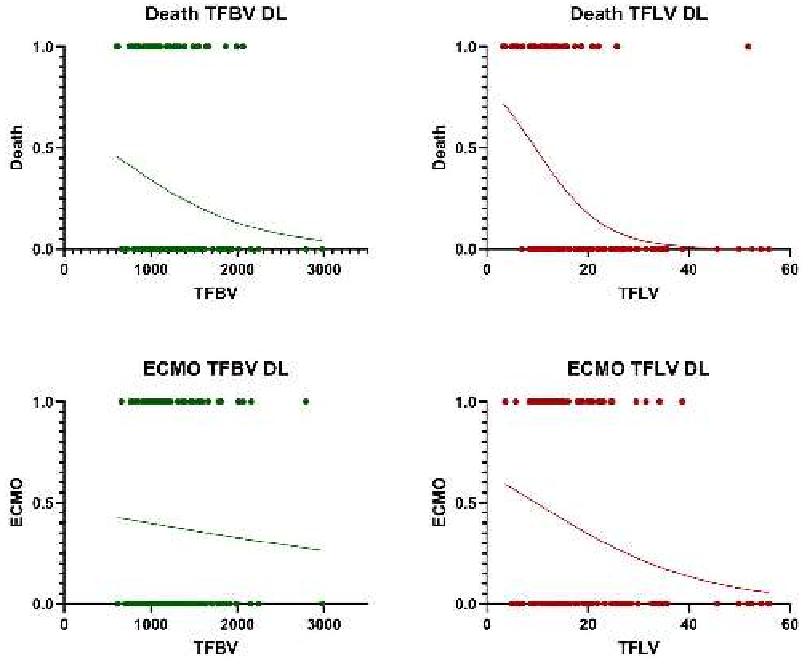

Figure S2: Correlation of deep learning (DL) segmented total fetal body volume (TFBV) and total fetal lung volume (TFLV) with death and extracorporeal membrane oxygenation (ECMO). With both higher TFBV and TFLV the probability of death and ECMO decreases.

Table S1: Acquisition parameters

Parameters	Balanced GRE		T2w TSE	
	**Minimum**	**Maximum**	**Minimum**	**Maximum**
Field of view (mm^2)	300 x 300	450 x 450	220 x 220	450 x 450
Acquisition image matrix	220 x 176	328 x 263	188 x 110	380 x 222
Acquired in-plane spatial resolution (mm^2)	1.17 x 1.17	2.00 x 1.76	1.36 x 0.89	2.67 x 1.56
Reconstruction image matrix	256 x 256	512 x 512	256 x 256	576 x 576
Reconstructed in-plane spatial resolution (mm^2)	0.88 x 0.88	1.76 x 1.76	0.65 x 0.65	1.17 x 1.17
Slice thickness (mm)	4.4	7	3	4.4
Echo time (ms)	1.59	4.68	80	158
Repetition time (ms)	3.18	9.37	1,440	27,301
Flip angle (°)	60	90	90	90

Note.—balanced GRE: balanced gradient echo sequence; T2w TSE: T2-weighted turbo spin echo sequence

Table S2: Results of the cross-validation dataset

Parameter		Number	Manual	DL	Absolute Difference	Relative difference (%)	P-value
TFBV (ml)	All	188 (100)	1213 ± 378	1212 ± 372	18.3 ± 27.1	1.5 ± 2.2	.82
	Polyhydramnios	70 (37)	1299 ± 382	1294 ± 376	17 ± 16	1.3 ± 1.2	.12
	Anhydramnios/ Oligohydramnios	4 (2)	1036 ± 445	1079 ± 464	59 ± 71	5.7 ± 6.9	.40
TFLV (ml)	All	188 (100)	17.5 ± 9.4	16.3 ± 8.8	2.2 ± 2.0	12.3 ± 11.6	<.001
	Liver-down	74 (39)	22.0 ± 9.4	20.6 ± 8.3	2.6 ± 1.8	11.8 ± 8.4	<.001
	Liver-up	114 (61)	14.6 ± 8.2	13.6 ± 8.0	1.9 ± 2.1	12.9 ± 14.5	<.001
	CDH right side	27 (14)	16.3 ± 11.1	14.2 ± 10.3	3.0 ± 3.6	18.6 ± 22.3	.02
	CDH left side	158 (84)	17.9 ± 9.0	16.9 ± 8.5	2.0 ± 1.6	11.3 ± 8.9	<.001
	CDH both sides	3 (2)	7.5 ± 6.1	5.5 ± 4.5	2.0 ± 1.6	26.0 ± 21.5	.17
	Lung parenchyma only right	11 (6)	10.9 ± 4.1	12.5 ± 4.5	1.9 ± 1.6	17.2 ± 15.0	.02
	Lung parenchyma only left	5 (3)	7.9 ± 2.9	3.7 ± 2.4	4.3 ± 3.7	54.4 ± 47.5	.08
	Lung parenchyma on both sides	172 (91)	18.2 ± 9.4	16.9 ± 8.8	2.1 ± 2.0	11.6 ± 10.9	<.001
O/E TLV (%)	All	188 (100)	38.3 ± 17.3	35.7 ± 16.5	4.6 ± 3.7	12.0 ± 9.8	<.001
	Polyhydramnios	70 (37)	35.3 ± 13.4	32.6 ± 13.0	4.7 ± 3.6	13.2 ± 10.3	<.001
	Anhydramnios/ Oligohydramnios	4 (2)	44.0 ± 14.0	32.3 ± 13.2	11.8 ± 5.3	26.7 ± 12.0	.02
	Liver-down	74 (39)	46.0 ± 15.1	43.1 ± 13.6	5.3 ± 3.8	11.4 ± 8.2	<.001
	Liver-up	114 (61)	33.3 ± 16.8	31.0 ± 16.4	4.2 ± 3.7	12.5 ± 11.1	<.001
	CDH right side	27 (14)	34.0 ± 26.7	30.4 ± 26.0	5.5 ± 5.4	16.1 ± 15.9	.01
	CDH left side	158 (84)	39.4 ± 15.0	37.1 ± 13.9	4.5 ± 3.4	11.3 ± 8.7	<.001
	CDH both sides	3 (2)	17.3 ± 6.2	12.7 ± 4.3	4.5 ± 1.9	26.3 ± 11.3	.06
	Lung parenchyma only right	11 (6)	23.8 ± 8.7	26.6 ± 8.3	4.1 ± 3.2	17.2 ± 13.6	.07
	Lung parenchyma only left	5 (3)	16.1 ± 3.8	9.0 ± 7.6	7.6 ± 6.6	47.1 ± 40.9	.09
	Lung parenchyma on both sides	172 (91)	39.8 ± 17.1	37.1 ± 16.2	4.5 ± 3.7	11.4 ± 9.2	<.001

Note.—The O/E TLV was calculated according to Cannie et al. (6).Continuous data is presented as mean ± standard deviation and categorical data as number of patients with percentages in parentheses. DL: deep learning; O/E TLV: observed/expected ratio of the total lung volume; TFBV: total fetal body volume; TFLV: total fetal lung volume.
